# Locoed Horses

**Published:** 1892-07

**Authors:** Frank A. McCullaugh


### LOCOED HORSES.

By Frank A. McCullaugh.

Few people living in the "States," unless they have visited the far west, or more correctly speaking the "plains," have ever heard of loco weed and its injurious effects upon all kinds of live stock. Veterinarians, as well as laymen, are equally ignorant as regards the pathological effects of the weed. I will, therefore, endeavor as briefly and concisely as possible to describe the disease and its symptoms, as it appeared to me, after having seen a very large number of locoed animals. The term loco, simply meaning foolish (or rather crazy), is applied because of the peculiar form of dementia induced in the animals that are in the habit of eating the plant. All herbivorous animals are liable to the disease, none are exempt, even antelopes have been known to be thus affected, but for the purposes of this article, the horse only will be considered. The discovery of the poisonous effects of the weed dates back many years, and was, as nearly as can be ascertained, first noticed west of the Rio Grande River. Since that time it has been carried by birds and the continuity of the wind, always to be found in the higher altitudes, to all parts of the western country, until its boundaries may be said to extend from the Upper Missouri on the north, the Lower Missouri and Mississippi on the east, south by Old Mexico and the Gulf, and west by California.

Having spoken in a general way of the weed and where it is to be found, we will now consider more minutely the effects, symptoms and termination. Its action is similar to that of alcohol, or some of the more powerful opiates used by mankind. In fact, I cannot compare a locoed horse better than to one of the human family who has fallen a victim to the morphine or opium habit. Loco becomes green in the early spring while the snow is yet on the ground, standing out clear and tempting to animals that have passed through the rigors and vicissitudes of a winter on the range. From personal knowledge, I do not think horses would, at first, eat the weed from choice, but are lured by its freshness before the grass has started. Even then it is not common for them to eat it, but having once started in the downward path of loco, a horse will abandon all forms of food and look for it. In a short time its effects become perfectly apparent. Among the symptoms first noticed are loss of flesh, general lassitude, and impaired vision. The last named, I think, might be taken as the first symptom of the disease, as I have always noticed the vision to be impaired before any other signs were visible.

The most gentle animal becomes a vicious shyer, and things he has been accustomed to see daily appear misshapen and of gigantic size. A shadow cast by a fence rail he will not cross, except he jumps high in the air, the movement destitute of grace. Falling backward and refusing to move when saddled is an early indication of the disease. About this time an exostosis appears on the middle and superior portion of the frontal bone, becoming at times as large as a pullet's egg, though generally half that size. It is permanent, and should the animal ultimately recover, the enlargement remains as a lasting blemish. An animal thus affected may easily be detected in the dark, and though a would-be-purchaser has all the light possible, it is always well to take the precaution of feeling for enlargements about the head, if there is any reason to think the animal has come from a loco region. Next we find the animal losing rapidly in flesh, until it reaches a state of hide bound, the coat is rough, and the unyielding skin will everywhere adhere close to the substance which it covers. In this condition an animal may continue to exist as long as from ten to eighteen months, if not a confirmed loco hunter; the latter seldom live to exceed three months. As the disease advances the animal loses all muscular power, tetanic symptoms appear, and if suddenly startled, will invariably fall. The expression is idiotic and extremely stupid. The muscles of the mouth and tongue become partially paralyzed, so that the prehension of food is impossible, mastication is commenced, but never completed. Thus the poor victim goes from day to day, becoming as weak physically as mentally, until death terminates its sufferings. I have many times offered water to locoed horses that I knew had not drank in five or six days, they would sometimes gaze stupidly for hours at a time at a trough filled with water, but make no attempt to drink; again, they would bite at the water as if masticating food, but could not complete the act of deglutition. From this it can readily be seen that the true cause of death is from starvation. In conclusion, I regret that I cannot give some practical or definite treatment for this prevalent disease, but the few experiments I have witnessed were unsuccessful, and any course of treatment

I might mention would be more or less of an empirical nature. However, if taken in the early stage and the cause removed, the animal fed liberally on nourishing food, there is a possible chance of recovery ; and if only partially so, the animal may still prove to be useful for harness purposes, but the result is uncertain, and unless the horse be a valuable one, the owner will find the experiment an expensive one.

